# Crystal structure of 1,8-dibenzoyl-2,7-di­phen­oxy­naphthalene

**DOI:** 10.1107/S1600536814019758

**Published:** 2014-09-06

**Authors:** Satsuki Narushima, Saki Mohri, Noriyuki Yonezawa, Akiko Okamoto

**Affiliations:** a Department of Organic and Polymer Materials Chemistry, Tokyo University of Agriculture & Technology (TUAT), Koganei, Tokyo 184-8588, Japan

**Keywords:** crystal structure, *peri*-aroyl­naphthalene, C—H⋯π inter­actions, spatial organization

## Abstract

In the title compound, C_36_H_24_O_4_, the benzene rings of the benzoyl and phen­oxy groups make dihedral angles of 75.01 (4), 75.78 (4), 83.17 (5) and 80.84 (5)° with the naphthalene ring system. In the crystal, two types of C—H⋯π inter­actions between the benzene rings of the benzoyl groups and the naphthalene unit, and two kinds of π–π inter­actions between the benzene rings, with centroid–centroid distances of 3.879 (1) and 3.696 (1) Å, are observed.

## Abstract 2

In 1,8-dibenzoyl-2,7-di­phen­oxy­naphthalene, the planes of the benzene rings of the four naphthalene substituents are almost perpendicular to that of the naphthalene core.

## Chemical context   

*Peri*-substituted naphthalenes have received much attention as characteristic-structured aromatic-ring-core compounds for a variety of functional materials (Mei *et al.*, 2006 ▶; Shinamura *et al.*, 2010 ▶; Jiang *et al.*, 2010 ▶; Shao *et al.*, 2014 ▶). For example, rylene derivatives are fluoro­phores well known for their exceptional photochemical stability and high fluorescence quantum yields (Würthner *et al.*, 2004 ▶; Jiao *et al.*, 2009 ▶), and employed in solar cells (Shibano *et al.*, 2009 ▶), laser dyes (Gvishi *et al.*, 1993 ▶), organic light-emitting field-effect trans­is­tors (Seo *et al.*, 2013 ▶) and optical switches (Oneil *et al.*, 1992 ▶). However, planar aromatic structures containing *peri*-substituted naphthalenes are prone to inter­molecular aggregation that often leads to serious problems including fluorescence quenching (Wang & Yu, 2010 ▶). Therefore, development of *peri*-substituted naphthalene derivatives with aromatic substituents twisted relative to the naphthalene ring system, to inhibit mol­ecular aggregation, has been desired. 
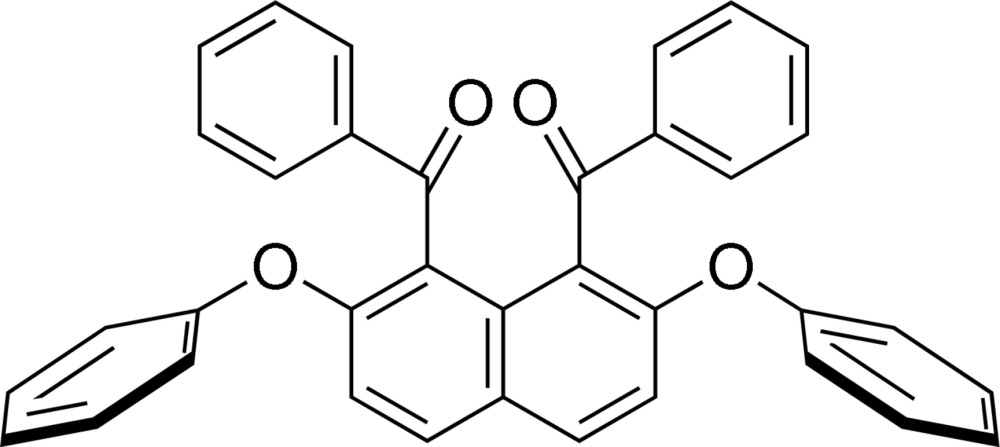


The authors have found that *peri*-aroyl­naphthalene compounds are afforded smoothly *via* electrophilic aromatic aroylation of a naphthalene derivative in the presence of a suitable acidic mediator (Okamoto & Yonezawa, 2009 ▶; Okamoto *et al.*, 2011 ▶). In *peri*-aroyl­naphthalene compounds, as a result of steric hindrance, the aroyl groups have to be arranged nearly perpendicular relative to the naphthalene core. Bearing this in mind, we have initiated a crystallographic study of *peri*-aroyl­naphthalene compounds in a search for correlation between the mol­ecular structure, the crystal packing and the non-bonding inter­actions (Okamoto *et al.*, 2014 ▶). Herein, the crystal structure of 1,8-dibenzoyl-2,7-di­phen­oxy­naphtahlene, (I), is reported and its structural features are discussed through comparison with the homologues, 1,8-bis­(4-fluoro­benzo­yl)-2,7-di­phen­oxy­naphthalene (Hijikata *et al.*, 2012 ▶) and 1,8-dibenzoyl-2,7-di­meth­oxy­naphthalene (Nakaema *et al.*, 2008 ▶).

## Structural commentary   

**Figure 1:**
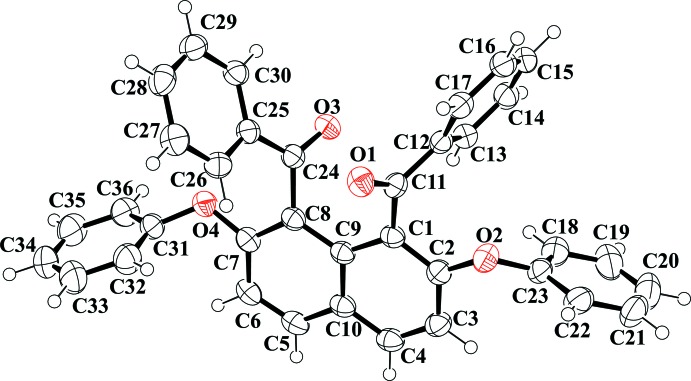
The mol­ecular structure of title mol­ecule, showing the atom-numbering scheme. Displacement ellipsoids are drawn at the 50% probability level.

The mol­ecular structure of (I) is displayed in Fig. 1 ▶. The benzene rings of the four substituents are arranged almost perpendicular relative to the naphthalene ring system. Furthermore, the two carbonyl groups attached at the 1- and 8-positions of the naphthalene ring are in the *anti* orientation. The benzene rings of the benzoyl groups make dihedral angles of 75.01 (4) and 75.78 (4)° with the naphthalene core. These dihedral angles are slightly smaller than those between the benzene rings of the phen­oxy groups at the 2- and 7-positions and the naphthalene ring [83.17 (5) and 80.84 (5)°]. The mol­ecular structure only slightly deviates from *C*_2_ symmetry and the mol­ecules exhibit axial chirality either with two *S*,*S* or two *R*,*R* stereogenic centers.

## Supra­molecular features   

**Figure 2:**
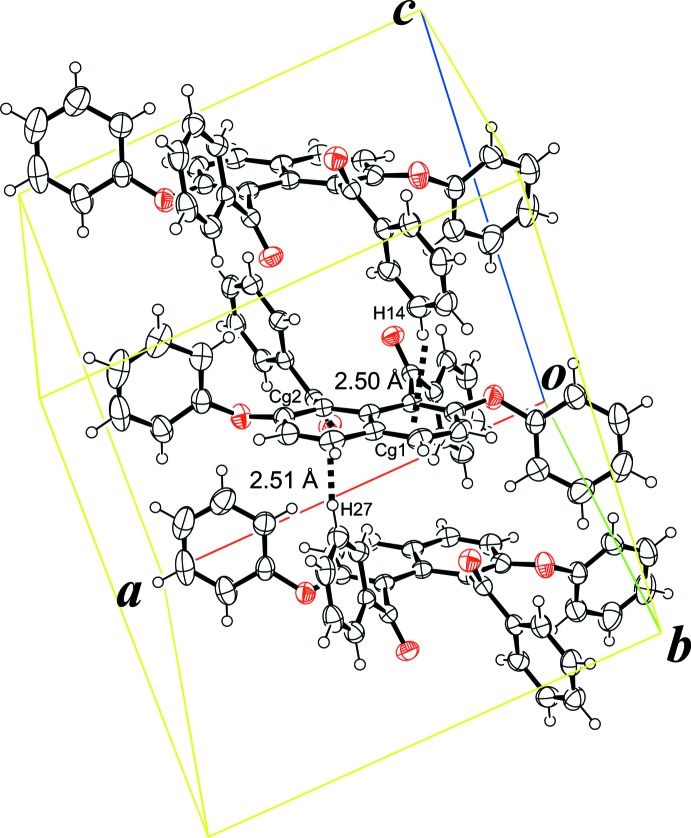
Two types of C—H⋯π inter­actions between the benzene ring of the benzoyl groups and the naphthalene rings, forming a single column structure (see Table 1 ▶ for details).

**Figure 3:**
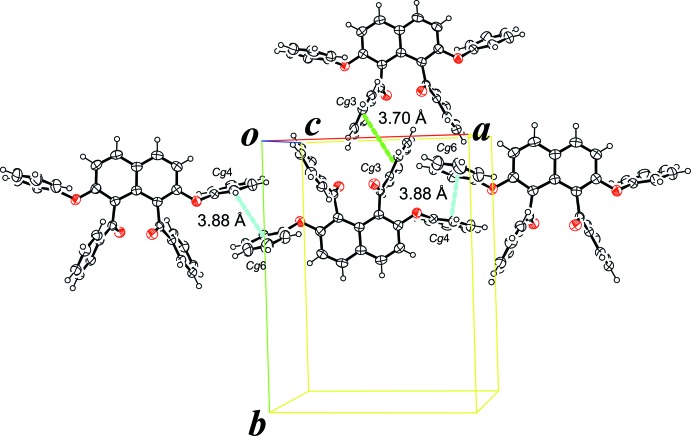
π–π inter­actions between the benzene rings of the benzoyl groups (green dashed line) and between the benzene rings of the phen­oxy groups (blue dashed lines).

In the crystal, *R*,*R* and *S*,*S*-isomers are alternately arranged along the *c* axis, forming a single column with the mol­ecules linked by two types of C—H⋯π inter­actions involving the benzene ring of the benzoyl groups and the naphthalene unit (Table 1 ▶ and Fig. 2 ▶). In addition, π–π stacking inter­actions are formed between mol­ecules in adjacent columns (Fig. 3 ▶). These inter­actions are observed between the benzene rings of the phen­oxy groups [*Cg*4 is the centroid of the C18–C23 ring and *Cg*6 is the centroid of the C31–C36 ring; *Cg4*⋯*Cg6*(*x* + 1, −*y* + 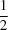, *z* + 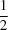) = 3.879 (1) Å] and the benzene rings of the benzoyl groups [*Cg*3 is the centroid of the C12–C17 ring; *Cg3*⋯C*g3*(−*x* + 1, −*y*, −*z* + 1) = 3.696 (1) Å].

## Database survey   

A search of the Cambridge Structural Database (Version 5.35, last update May 2014; Allen, 2002 ▶) showed 39 structures of 1,8-diaroyl­naphthalenes and 1,8-dialkanoyl­naphthalenes and 30 structures of 1,8-diaroyl-2,7-di­alk­oxy­naphthalenes and 1,8-diaroyl-2,7-di­aryl­oxynaphthalenes. The title compound, (I), is closely related to 1,8-bis­(4-fluoro­benzo­yl)-2,7-di­phen­oxy­naphthalene, (II) (Hijikata *et al.*, 2012 ▶), and 1,8-dibenzoyl-2,7-di­meth­oxy­naphthalene, (III) (Nakaema *et al.*, 2008 ▶). Like in the title compound, in homologue (II), the four benzene rings are non-coplanarly oriented relative to the naphthalene core. The dihedral angles formed by the benzene rings of the benzoyl groups are very similar to the title compound (I) [72.07 (4) and 73.24 (4)°], whereas those of the benzene rings of the phen­oxy groups differ and are both smaller than in the title compound [62.49 (5) and 77.96 (5)°]. Homologue (III) is apparently different as the mol­ecule is located on a crystallographic twofold rotation axis passing through the two central C atoms of the naphthalene unit. The dihedral angle between the benzene ring of the benzoyl group and the naphthalene ring system is 80.25 (6)°. In homologues (II) and (III), the mol­ecules are linked by (*sp* ^2^)C—H⋯O=C hydrogen bonds, forming a column structure [H⋯O = 2.40 Å for homologue (II) and 2.60 Å for homologue (III)]. In homologue (II), C—H⋯π inter­actions between the benzene ring of the benzoyl group and the benzene ring of the phen­oxy group (2.80 Å) are observed. In homologue (III), π–π inter­actions between the benzene rings of the benzoyl groups are formed [centroid–centroid and inter­planar distances of 3.6383 (10) and 3.294 Å, respectively]. On the other hand, the title structure forms no C—H⋯O=C inter­actions shorter than 2.70 Å. In (I), C—H⋯π and π–π stacking inter­actions evidently predominate.

## Synthesis and crystallization   

1,8-Dibenzoyl-2,7-di­hydroxy­naphthalene (0.2 mmol, 74 mg), benzenboronic acid (0.8 mmol, 97 mg), Cu(OAc)_2_ (0.4 mmol, 73 mg), activated 4 Å mol­ecular sieves (0.2 g), pyridine (1.6 mmol, 126 mg) and methyl­ene chloride (0.8 ml) were placed in a 10 ml flask. The reaction mixture was stirred at room temperature for 48 h and then diluted with CHCl_3_ (10 ml). The solution was successively washed with saturated aqueous NH_4_Cl, 2*M* aqueous HCl and brine. The organic layers thus obtained were dried over anhydrous MgSO_4_. After removal of solvent under reduced pressure, the crude product was purified by column chromatography (silica gel, hexa­ne–AcOEt, 2:1 *v*/*v*; isolated yield 68%). The isolated product was crystallized from ethanol to give single crystals.

^1^H NMR (300 MHz, CDCl_3_): δ 6.82 (4H, *d*, *J* = 8.4 Hz), 7.03 (2H, *t*, *J* = 7.2 Hz), 7.08 (2H, *d*, *J* = 9.3 Hz), 7.22 (4H, *t*, *J* = 7.5 Hz), 7.33 (4H, *t*, *J* = 7.8 Hz), 7.46 (2H, *t*, *J* = 6.9 Hz), 7.80 (4H, *d*, *J* = 7.5 Hz), 7.89 (2H, *d*, *J* = 9.0 Hz); ^13^C NMR (75 MHz, CDCl_3_): δ 117.333, 119.169, 123.863, 125.374, 127.984, 128.070, 129.361, 129.714, 131.980, 133.022, 138.501, 153.884, 156.121, 179.239, 196.142; IR (KBr): ν 1655, 1614, 1592, 1504 cm^−1^; HRMS (*m*/*z*): [*M*+H]^+^ calculated for C_30_H_25_O_4_, 521.1753; found, 521.1768; m.p. 423.6–424.4 K. 

## Refinement details   

Crystal data, data collection and structure refinement details are summarized in Table 2 ▶. All H atoms were located in a difference Fourier map and were subsequently refined as riding on their carriers, with C—H = 0.95 Å (aromatic) and *U*_iso_(H) = 1.2 *U*_eq_(C).

## Supplementary Material

Crystal structure: contains datablock(s) I. DOI: 10.1107/S1600536814019758/gk2618sup1.cif

Structure factors: contains datablock(s) I. DOI: 10.1107/S1600536814019758/gk2618Isup2.hkl

Supporting information file. DOI: 10.1107/S1600536814019758/gk2618Isup3.pdf

Supporting information file. DOI: 10.1107/S1600536814019758/gk2618Isup4.pdf

Supporting information file. DOI: 10.1107/S1600536814019758/gk2618Isup5.pdf

Supporting information file. DOI: 10.1107/S1600536814019758/gk2618Isup6.pdf

Supporting information file. DOI: 10.1107/S1600536814019758/gk2618Isup7.cml

CCDC reference: 1022493

Additional supporting information:  crystallographic information; 3D view; checkCIF report

## Figures and Tables

**Table 1:** Hydrogen-bond geometry (Å, °) *Cg*1 and *Cg*2 are the centroids of the C1–C4/C10/C9 and C5–C10 rings, respectively.

*D*—H⋯*A*	*D*—H	H⋯*A*	*D*⋯*A*	*D*—H⋯*A*
C14—H14⋯*Cg*1^i^	0.95	2.50	3.4192 (12)	163
C27—H27⋯*Cg*2^ii^	0.95	2.51	3.4002 (12)	155

Symmetry codes: (i) 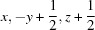; (ii) 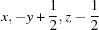.

**Table 2:** Experimental details

Crystal data	
Chemical formula	C_36_H_24_O_4_
*M*_r_	520.55
Crystal system, space group	Monoclinic, *P*2_1_/*c*
Temperature (K)	193
*a*, *b*, *c* (Å)	12.7734 (2), 16.4106 (3), 12.9012 (2)
β (°)	95.939 (1)
*V* (Å^3^)	2689.81 (9)
*Z*	4
Radiation type	Cu *K*α
μ (mm^−1^)	0.66
Crystal size (mm)	0.50 × 0.35 × 0.10

Data collection	
Diffractometer	Rigaku R-AXIS RAPID
Absorption correction	Numerical (*NUMABS*; Higashi, 1999 ▶)
*T*_min_, *T*_max_	0.732, 0.937
No. of measured, independent and observed [*I* > 2σ(*I*)] reflections	49716, 4924, 4506
*R*_int_	0.041
(sin θ/λ)_max_ (Å^−1^)	0.602

Refinement	
*R*[*F* ^2^ > 2σ(*F* ^2^)], *wR*(*F* ^2^), *S*	0.034, 0.097, 1.04
No. of reflections	4924
No. of parameters	362
H-atom treatment	H-atom parameters constrained
Δρ_max_, Δρ_min_ (e Å^−3^)	0.21, −0.19

Computer programs: *PROCESS-AUTO* (Rigaku, 1998 ▶), *CrystalStructure* (Rigaku, 2007 ▶), *SIR2004* (Burla *et al.*, 2007 ▶), *SHELXL97* (Sheldrick, 2008 ▶) and *ORTEPIII* (Burnett & Johnson, 1996 ▶).

## supplementary crystallographic information

### Crystal data

**Table:**

C_36_H_24_O_4_	*F*(000) = 1088
*M**_r_* = 520.55	*D*_x_ = 1.285 Mg m^−^^3^
Monoclinic, *P*2_1_/*c*	Cu *K*α radiation, λ = 1.54187 Å
Hall symbol: -P 2ybc	Cell parameters from 26940 reflections
*a* = 12.7734 (2) Å	θ = 3.4–68.2°
*b* = 16.4106 (3) Å	µ = 0.66 mm^−^^1^
*c* = 12.9012 (2) Å	*T* = 193 K
β = 95.939 (1)°	Platelet, colorless
*V* = 2689.81 (9) Å^3^	0.50 × 0.35 × 0.10 mm
*Z* = 4	

### Data collection

**Table:**

Rigaku R-AXIS RAPID diffractometer	4924 independent reflections
Radiation source: fine-focus sealed tube	4506 reflections with *I* > 2σ(*I*)
Graphite monochromator	*R*_int_ = 0.041
Detector resolution: 10.000 pixels mm^-1^	θ_max_ = 68.2°, θ_min_ = 3.5°
ω scans	*h* = −15→14
Absorption correction: numerical (*NUMABS*; Higashi, 1999)	*k* = −19→19
*T*_min_ = 0.732, *T*_max_ = 0.937	*l* = −15→15
49716 measured reflections	

### Refinement

**Table:**

Refinement on *F*^2^	Secondary atom site location: difference Fourier map
Least-squares matrix: full	Hydrogen site location: inferred from neighbouring sites
*R*[*F*^2^ > 2σ(*F*^2^)] = 0.034	H-atom parameters constrained
*wR*(*F*^2^) = 0.097	*w* = 1/[σ^2^(*F*_o_^2^) + (0.0558*P*)^2^ + 0.4291*P*] where *P* = (*F*_o_^2^ + 2*F*_c_^2^)/3
*S* = 1.04	(Δ/σ)_max_ < 0.001
4924 reflections	Δρ_max_ = 0.21 e Å^−^^3^
362 parameters	Δρ_min_ = −0.19 e Å^−^^3^
0 restraints	Extinction correction: *SHELXL97* (Sheldrick, 2008), Fc^*^=kFc[1+0.001xFc^2^λ^3^/sin(2θ)]^-1/4^
Primary atom site location: structure-invariant direct methods	Extinction coefficient: 0.00272 (19)

### Special details

**Table:**

Geometry. All e.s.d.'s (except the e.s.d. in the dihedral angle between two l.s. planes) are estimated using the full covariance matrix. The cell e.s.d.'s are taken into account individually in the estimation of e.s.d.'s in distances, angles and torsion angles; correlations between e.s.d.'s in cell parameters are only used when they are defined by crystal symmetry. An approximate (isotropic) treatment of cell e.s.d.'s is used for estimating e.s.d.'s involving l.s. planes.
Refinement. Refinement of *F*^2^ against ALL reflections. The weighted *R*-factor *wR* and goodness of fit *S* are based on *F*^2^, conventional *R*-factors *R* are based on *F*, with *F* set to zero for negative *F*^2^. The threshold expression of *F*^2^ > σ(*F*^2^) is used only for calculating *R*-factors(gt) *etc*. and is not relevant to the choice of reflections for refinement. *R*-factors based on *F*^2^ are statistically about twice as large as those based on *F*, and *R*- factors based on ALL data will be even larger.

### Fractional atomic coordinates and isotropic or equivalent isotropic displacement parameters (Å^2^)

**Table:**

	*x*	*y*	*z*	*U*_iso_*/*U*_eq_	
O1	0.50123 (6)	0.17091 (5)	0.26210 (6)	0.0420 (2)	
O2	0.68204 (6)	0.28487 (5)	0.43151 (6)	0.0423 (2)	
O3	0.29940 (6)	0.17736 (5)	0.40091 (6)	0.04128 (19)	
O4	0.13200 (6)	0.30376 (5)	0.23233 (6)	0.0453 (2)	
C1	0.50205 (8)	0.28673 (6)	0.37033 (7)	0.0324 (2)	
C2	0.59047 (8)	0.32915 (7)	0.40983 (8)	0.0357 (2)	
C3	0.59303 (9)	0.41494 (7)	0.41644 (8)	0.0410 (3)	
H3	0.6547	0.4423	0.4456	0.049*	
C4	0.50574 (9)	0.45778 (7)	0.38032 (8)	0.0400 (3)	
H4	0.5076	0.5156	0.3823	0.048*	
C5	0.32265 (9)	0.46409 (6)	0.30298 (8)	0.0401 (3)	
H5	0.3258	0.5218	0.3075	0.048*	
C6	0.23228 (9)	0.42820 (7)	0.26128 (8)	0.0417 (3)	
H6	0.1739	0.4603	0.2341	0.050*	
C7	0.22675 (8)	0.34215 (7)	0.25911 (8)	0.0372 (2)	
C8	0.31057 (8)	0.29366 (6)	0.29617 (7)	0.0332 (2)	
C9	0.40819 (8)	0.33102 (6)	0.33549 (7)	0.0329 (2)	
C10	0.41238 (8)	0.41796 (6)	0.33991 (7)	0.0358 (2)	
C11	0.51520 (7)	0.19679 (6)	0.35110 (8)	0.0322 (2)	
C12	0.54959 (7)	0.14356 (6)	0.44108 (8)	0.0317 (2)	
C13	0.53226 (8)	0.16529 (6)	0.54194 (8)	0.0356 (2)	
H13	0.4984	0.2154	0.5544	0.043*	
C14	0.56439 (9)	0.11396 (7)	0.62452 (9)	0.0426 (3)	
H14	0.5524	0.1289	0.6934	0.051*	
C15	0.61378 (8)	0.04119 (7)	0.60686 (10)	0.0455 (3)	
H15	0.6352	0.0061	0.6636	0.055*	
C16	0.63217 (8)	0.01926 (7)	0.50689 (10)	0.0450 (3)	
H16	0.6671	−0.0305	0.4951	0.054*	
C17	0.59953 (8)	0.06985 (6)	0.42409 (9)	0.0384 (2)	
H17	0.6112	0.0543	0.3553	0.046*	
C18	0.75171 (8)	0.30897 (6)	0.51655 (9)	0.0409 (3)	
C19	0.85574 (10)	0.31908 (8)	0.50004 (12)	0.0541 (3)	
H19	0.8777	0.3125	0.4324	0.065*	
C20	0.92813 (11)	0.33918 (9)	0.58451 (15)	0.0703 (5)	
H20	1.0000	0.3470	0.5743	0.084*	
C21	0.89623 (12)	0.34786 (9)	0.68263 (14)	0.0722 (5)	
H21	0.9460	0.3617	0.7398	0.087*	
C22	0.79282 (12)	0.33645 (8)	0.69766 (11)	0.0630 (4)	
H22	0.7712	0.3418	0.7656	0.076*	
C23	0.71910 (10)	0.31718 (7)	0.61457 (10)	0.0485 (3)	
H23	0.6472	0.3098	0.6251	0.058*	
C24	0.28832 (8)	0.20438 (6)	0.31252 (8)	0.0335 (2)	
C25	0.24927 (8)	0.15255 (6)	0.22211 (8)	0.0336 (2)	
C26	0.26596 (8)	0.17354 (7)	0.12080 (8)	0.0399 (2)	
H26	0.3020	0.2225	0.1078	0.048*	
C27	0.22996 (9)	0.12298 (7)	0.03865 (9)	0.0467 (3)	
H27	0.2418	0.1374	−0.0305	0.056*	
C28	0.17703 (9)	0.05188 (7)	0.05675 (10)	0.0479 (3)	
H28	0.1523	0.0176	0.0001	0.057*	
C29	0.15999 (9)	0.03062 (7)	0.15734 (10)	0.0468 (3)	
H29	0.1233	−0.0182	0.1697	0.056*	
C30	0.19624 (8)	0.08026 (7)	0.23989 (9)	0.0394 (2)	
H30	0.1850	0.0652	0.3090	0.047*	
C31	0.06162 (8)	0.33949 (7)	0.15531 (9)	0.0404 (3)	
C32	0.09041 (10)	0.35598 (9)	0.05780 (10)	0.0531 (3)	
H32	0.1604	0.3465	0.0419	0.064*	
C33	0.01505 (12)	0.38679 (10)	−0.01674 (11)	0.0679 (4)	
H33	0.0339	0.3994	−0.0842	0.082*	
C34	−0.08662 (11)	0.39936 (10)	0.00530 (12)	0.0675 (4)	
H34	−0.1377	0.4199	−0.0469	0.081*	
C35	−0.11407 (10)	0.38220 (9)	0.10278 (13)	0.0616 (4)	
H35	−0.1844	0.3908	0.1182	0.074*	
C36	−0.03952 (9)	0.35228 (8)	0.17909 (10)	0.0486 (3)	
H36	−0.0581	0.3408	0.2470	0.058*	

### Atomic displacement parameters (Å^2^)

**Table:**

	*U*^11^	*U*^22^	*U*^33^	*U*^12^	*U*^13^	*U*^23^
O1	0.0491 (4)	0.0440 (4)	0.0323 (4)	0.0028 (3)	0.0023 (3)	−0.0061 (3)
O2	0.0355 (4)	0.0421 (4)	0.0487 (4)	−0.0006 (3)	0.0015 (3)	−0.0073 (3)
O3	0.0456 (4)	0.0421 (4)	0.0358 (4)	−0.0013 (3)	0.0026 (3)	0.0084 (3)
O4	0.0368 (4)	0.0427 (4)	0.0551 (5)	0.0022 (3)	−0.0015 (3)	0.0100 (4)
C1	0.0378 (5)	0.0323 (5)	0.0276 (5)	−0.0010 (4)	0.0056 (4)	0.0017 (4)
C2	0.0369 (5)	0.0379 (6)	0.0324 (5)	−0.0011 (4)	0.0048 (4)	−0.0001 (4)
C3	0.0449 (6)	0.0389 (6)	0.0388 (6)	−0.0084 (5)	0.0028 (5)	−0.0033 (4)
C4	0.0546 (7)	0.0305 (5)	0.0352 (5)	−0.0033 (5)	0.0064 (5)	−0.0014 (4)
C5	0.0553 (7)	0.0307 (5)	0.0350 (5)	0.0050 (5)	0.0069 (5)	0.0033 (4)
C6	0.0477 (6)	0.0373 (6)	0.0395 (6)	0.0095 (5)	0.0019 (5)	0.0065 (4)
C7	0.0396 (6)	0.0387 (6)	0.0334 (5)	0.0024 (4)	0.0039 (4)	0.0033 (4)
C8	0.0379 (5)	0.0329 (5)	0.0291 (5)	0.0018 (4)	0.0048 (4)	0.0015 (4)
C9	0.0394 (6)	0.0325 (5)	0.0273 (5)	0.0008 (4)	0.0056 (4)	0.0019 (4)
C10	0.0467 (6)	0.0328 (5)	0.0285 (5)	0.0003 (4)	0.0066 (4)	0.0011 (4)
C11	0.0282 (5)	0.0361 (5)	0.0327 (5)	−0.0010 (4)	0.0049 (4)	−0.0026 (4)
C12	0.0280 (5)	0.0307 (5)	0.0363 (5)	−0.0020 (4)	0.0023 (4)	−0.0008 (4)
C13	0.0357 (5)	0.0350 (5)	0.0362 (5)	−0.0006 (4)	0.0043 (4)	−0.0003 (4)
C14	0.0404 (6)	0.0497 (7)	0.0373 (6)	−0.0047 (5)	0.0016 (4)	0.0056 (5)
C15	0.0339 (5)	0.0459 (6)	0.0545 (7)	−0.0030 (5)	−0.0060 (5)	0.0162 (5)
C16	0.0323 (5)	0.0335 (5)	0.0681 (8)	0.0029 (4)	0.0000 (5)	0.0040 (5)
C17	0.0333 (5)	0.0350 (5)	0.0470 (6)	0.0004 (4)	0.0043 (4)	−0.0047 (4)
C18	0.0352 (5)	0.0317 (5)	0.0541 (7)	−0.0020 (4)	−0.0038 (5)	−0.0007 (5)
C19	0.0393 (6)	0.0459 (7)	0.0767 (9)	−0.0044 (5)	0.0044 (6)	0.0080 (6)
C20	0.0355 (7)	0.0543 (8)	0.1166 (14)	−0.0062 (6)	−0.0134 (7)	0.0059 (8)
C21	0.0621 (9)	0.0542 (8)	0.0917 (12)	0.0063 (7)	−0.0322 (8)	−0.0148 (8)
C22	0.0722 (9)	0.0498 (7)	0.0619 (8)	0.0152 (7)	−0.0172 (7)	−0.0131 (6)
C23	0.0466 (7)	0.0427 (6)	0.0548 (7)	0.0038 (5)	−0.0010 (5)	−0.0077 (5)
C24	0.0293 (5)	0.0358 (5)	0.0358 (5)	0.0022 (4)	0.0052 (4)	0.0042 (4)
C25	0.0292 (5)	0.0326 (5)	0.0390 (5)	0.0034 (4)	0.0029 (4)	0.0020 (4)
C26	0.0401 (6)	0.0396 (6)	0.0408 (6)	−0.0016 (4)	0.0072 (4)	0.0014 (4)
C27	0.0496 (6)	0.0513 (7)	0.0396 (6)	0.0037 (5)	0.0066 (5)	−0.0045 (5)
C28	0.0446 (6)	0.0435 (6)	0.0536 (7)	0.0041 (5)	−0.0043 (5)	−0.0107 (5)
C29	0.0413 (6)	0.0354 (6)	0.0622 (7)	−0.0021 (5)	−0.0019 (5)	0.0004 (5)
C30	0.0348 (5)	0.0362 (5)	0.0468 (6)	0.0018 (4)	0.0024 (4)	0.0066 (4)
C31	0.0362 (6)	0.0358 (5)	0.0479 (6)	0.0041 (4)	−0.0012 (4)	0.0016 (5)
C32	0.0428 (6)	0.0652 (8)	0.0512 (7)	−0.0024 (6)	0.0052 (5)	0.0040 (6)
C33	0.0655 (9)	0.0857 (11)	0.0499 (8)	−0.0132 (8)	−0.0071 (6)	0.0131 (7)
C34	0.0547 (8)	0.0672 (9)	0.0744 (10)	−0.0014 (7)	−0.0229 (7)	0.0138 (7)
C35	0.0385 (6)	0.0567 (8)	0.0881 (10)	0.0112 (6)	−0.0015 (6)	−0.0009 (7)
C36	0.0423 (6)	0.0471 (7)	0.0571 (7)	0.0076 (5)	0.0079 (5)	−0.0018 (5)

### Geometric parameters (Å, º)

**Table:**

O1—C11	1.2199 (12)	C18—C19	1.3774 (17)
O2—C2	1.3808 (13)	C18—C23	1.3784 (17)
O2—C18	1.3964 (13)	C19—C20	1.394 (2)
O3—C24	1.2181 (12)	C19—H19	0.9500
O4—C7	1.3763 (13)	C20—C21	1.377 (2)
O4—C31	1.3979 (13)	C20—H20	0.9500
C1—C2	1.3786 (14)	C21—C22	1.368 (2)
C1—C9	1.4338 (14)	C21—H21	0.9500
C1—C11	1.5090 (14)	C22—C23	1.3882 (17)
C2—C3	1.4107 (15)	C22—H22	0.9500
C3—C4	1.3590 (16)	C23—H23	0.9500
C3—H3	0.9500	C24—C25	1.4873 (14)
C4—C10	1.4115 (15)	C25—C26	1.3893 (15)
C4—H4	0.9500	C25—C30	1.3967 (15)
C5—C6	1.3566 (16)	C26—C27	1.3863 (16)
C5—C10	1.4145 (15)	C26—H26	0.9500
C5—H5	0.9500	C27—C28	1.3805 (17)
C6—C7	1.4141 (16)	C27—H27	0.9500
C6—H6	0.9500	C28—C29	1.3827 (18)
C7—C8	1.3791 (14)	C28—H28	0.9500
C8—C9	1.4343 (14)	C29—C30	1.3821 (16)
C8—C24	1.5115 (14)	C29—H29	0.9500
C9—C10	1.4286 (15)	C30—H30	0.9500
C11—C12	1.4826 (14)	C31—C32	1.3736 (17)
C12—C13	1.3889 (14)	C31—C36	1.3744 (16)
C12—C17	1.3952 (14)	C32—C33	1.3840 (19)
C13—C14	1.3866 (15)	C32—H32	0.9500
C13—H13	0.9500	C33—C34	1.374 (2)
C14—C15	1.3803 (17)	C33—H33	0.9500
C14—H14	0.9500	C34—C35	1.369 (2)
C15—C16	1.3823 (18)	C34—H34	0.9500
C15—H15	0.9500	C35—C36	1.3868 (18)
C16—C17	1.3827 (16)	C35—H35	0.9500
C16—H16	0.9500	C36—H36	0.9500
C17—H17	0.9500		
			
C2—O2—C18	117.89 (8)	C23—C18—O2	121.33 (10)
C7—O4—C31	118.07 (8)	C18—C19—C20	118.69 (14)
C2—C1—C9	119.10 (9)	C18—C19—H19	120.7
C2—C1—C11	116.98 (9)	C20—C19—H19	120.7
C9—C1—C11	123.23 (9)	C21—C20—C19	120.52 (14)
C1—C2—O2	116.98 (9)	C21—C20—H20	119.7
C1—C2—C3	122.63 (10)	C19—C20—H20	119.7
O2—C2—C3	119.94 (9)	C22—C21—C20	119.89 (13)
C4—C3—C2	118.85 (10)	C22—C21—H21	120.1
C4—C3—H3	120.6	C20—C21—H21	120.1
C2—C3—H3	120.6	C21—C22—C23	120.64 (15)
C3—C4—C10	121.26 (10)	C21—C22—H22	119.7
C3—C4—H4	119.4	C23—C22—H22	119.7
C10—C4—H4	119.4	C18—C23—C22	119.05 (13)
C6—C5—C10	121.86 (10)	C18—C23—H23	120.5
C6—C5—H5	119.1	C22—C23—H23	120.5
C10—C5—H5	119.1	O3—C24—C25	121.54 (9)
C5—C6—C7	118.69 (10)	O3—C24—C8	118.57 (9)
C5—C6—H6	120.7	C25—C24—C8	119.84 (8)
C7—C6—H6	120.7	C26—C25—C30	119.29 (10)
O4—C7—C8	117.00 (9)	C26—C25—C24	121.63 (9)
O4—C7—C6	120.19 (9)	C30—C25—C24	119.07 (9)
C8—C7—C6	122.27 (10)	C27—C26—C25	119.99 (10)
C7—C8—C9	119.43 (9)	C27—C26—H26	120.0
C7—C8—C24	117.21 (9)	C25—C26—H26	120.0
C9—C8—C24	122.28 (9)	C28—C27—C26	120.40 (11)
C10—C9—C1	117.84 (9)	C28—C27—H27	119.8
C10—C9—C8	117.94 (9)	C26—C27—H27	119.8
C1—C9—C8	124.22 (9)	C27—C28—C29	119.96 (11)
C4—C10—C5	120.05 (10)	C27—C28—H28	120.0
C4—C10—C9	120.25 (9)	C29—C28—H28	120.0
C5—C10—C9	119.70 (10)	C30—C29—C28	120.12 (11)
O1—C11—C12	122.31 (9)	C30—C29—H29	119.9
O1—C11—C1	119.17 (9)	C28—C29—H29	119.9
C12—C11—C1	118.47 (8)	C29—C30—C25	120.24 (10)
C13—C12—C17	119.31 (9)	C29—C30—H30	119.9
C13—C12—C11	121.28 (9)	C25—C30—H30	119.9
C17—C12—C11	119.41 (9)	C32—C31—C36	121.41 (11)
C14—C13—C12	120.04 (10)	C32—C31—O4	121.35 (10)
C14—C13—H13	120.0	C36—C31—O4	117.10 (10)
C12—C13—H13	120.0	C31—C32—C33	118.39 (12)
C15—C14—C13	120.18 (11)	C31—C32—H32	120.8
C15—C14—H14	119.9	C33—C32—H32	120.8
C13—C14—H14	119.9	C34—C33—C32	121.04 (14)
C14—C15—C16	120.26 (10)	C34—C33—H33	119.5
C14—C15—H15	119.9	C32—C33—H33	119.5
C16—C15—H15	119.9	C35—C34—C33	119.78 (12)
C15—C16—C17	119.84 (10)	C35—C34—H34	120.1
C15—C16—H16	120.1	C33—C34—H34	120.1
C17—C16—H16	120.1	C34—C35—C36	120.16 (13)
C16—C17—C12	120.36 (10)	C34—C35—H35	119.9
C16—C17—H17	119.8	C36—C35—H35	119.9
C12—C17—H17	119.8	C31—C36—C35	119.21 (13)
C19—C18—C23	121.21 (11)	C31—C36—H36	120.4
C19—C18—O2	117.33 (11)	C35—C36—H36	120.4
			
C9—C1—C2—O2	−172.98 (8)	C11—C12—C13—C14	179.40 (9)
C11—C1—C2—O2	−2.16 (13)	C12—C13—C14—C15	0.07 (16)
C9—C1—C2—C3	−0.74 (15)	C13—C14—C15—C16	0.44 (16)
C11—C1—C2—C3	170.07 (9)	C14—C15—C16—C17	−0.98 (16)
C18—O2—C2—C1	−145.91 (9)	C15—C16—C17—C12	1.01 (16)
C18—O2—C2—C3	41.63 (13)	C13—C12—C17—C16	−0.51 (15)
C1—C2—C3—C4	−1.54 (16)	C11—C12—C17—C16	−179.95 (9)
O2—C2—C3—C4	170.48 (9)	C2—O2—C18—C19	−129.66 (11)
C2—C3—C4—C10	2.13 (16)	C2—O2—C18—C23	54.45 (14)
C10—C5—C6—C7	2.80 (16)	C23—C18—C19—C20	−0.98 (18)
C31—O4—C7—C8	−151.93 (10)	O2—C18—C19—C20	−176.87 (11)
C31—O4—C7—C6	36.35 (14)	C18—C19—C20—C21	0.7 (2)
C5—C6—C7—O4	170.07 (9)	C19—C20—C21—C22	0.2 (2)
C5—C6—C7—C8	−1.19 (16)	C20—C21—C22—C23	−0.8 (2)
O4—C7—C8—C9	−173.51 (8)	C19—C18—C23—C22	0.36 (18)
C6—C7—C8—C9	−1.98 (15)	O2—C18—C23—C22	176.10 (11)
O4—C7—C8—C24	−5.11 (14)	C21—C22—C23—C18	0.6 (2)
C6—C7—C8—C24	166.42 (10)	C7—C8—C24—O3	−114.72 (11)
C2—C1—C9—C10	2.35 (13)	C9—C8—C24—O3	53.32 (14)
C11—C1—C9—C10	−167.86 (9)	C7—C8—C24—C25	62.62 (13)
C2—C1—C9—C8	−177.90 (9)	C9—C8—C24—C25	−129.33 (10)
C11—C1—C9—C8	11.89 (14)	O3—C24—C25—C26	−160.13 (10)
C7—C8—C9—C10	3.46 (14)	C8—C24—C25—C26	22.61 (14)
C24—C8—C9—C10	−164.32 (9)	O3—C24—C25—C30	18.66 (15)
C7—C8—C9—C1	−176.29 (9)	C8—C24—C25—C30	−158.60 (9)
C24—C8—C9—C1	15.92 (14)	C30—C25—C26—C27	0.10 (16)
C3—C4—C10—C5	179.91 (10)	C24—C25—C26—C27	178.89 (10)
C3—C4—C10—C9	−0.47 (15)	C25—C26—C27—C28	0.31 (17)
C6—C5—C10—C4	178.39 (10)	C26—C27—C28—C29	−0.23 (18)
C6—C5—C10—C9	−1.23 (15)	C27—C28—C29—C30	−0.26 (17)
C1—C9—C10—C4	−1.79 (14)	C28—C29—C30—C25	0.68 (17)
C8—C9—C10—C4	178.44 (9)	C26—C25—C30—C29	−0.59 (15)
C1—C9—C10—C5	177.84 (8)	C24—C25—C30—C29	−179.41 (9)
C8—C9—C10—C5	−1.94 (14)	C7—O4—C31—C32	56.41 (15)
C2—C1—C11—O1	−115.66 (11)	C7—O4—C31—C36	−127.86 (11)
C9—C1—C11—O1	54.74 (13)	C36—C31—C32—C33	0.6 (2)
C2—C1—C11—C12	61.54 (12)	O4—C31—C32—C33	176.12 (12)
C9—C1—C11—C12	−128.06 (10)	C31—C32—C33—C34	−1.1 (2)
O1—C11—C12—C13	−158.43 (10)	C32—C33—C34—C35	0.8 (2)
C1—C11—C12—C13	24.46 (14)	C33—C34—C35—C36	0.1 (2)
O1—C11—C12—C17	21.00 (14)	C32—C31—C36—C35	0.24 (19)
C1—C11—C12—C17	−156.11 (9)	O4—C31—C36—C35	−175.48 (11)
C17—C12—C13—C14	−0.03 (15)	C34—C35—C36—C31	−0.6 (2)

### Hydrogen-bond geometry (Å, º)

Cg1 and Cg2 are the centroids of the C1–C4/C10/C9 and C5–C10 rings, respectively.

**Table:**

*D*—H···*A*	*D*—H	H···*A*	*D*···*A*	*D*—H···*A*
C14—H14···*Cg*1^i^	0.95	2.50	3.4192 (12)	163
C27—H27···*Cg*2^ii^	0.95	2.51	3.4002 (12)	155

Symmetry codes: (i) *x*, −*y*+1/2, *z*+1/2; (ii) *x*, −*y*+1/2, *z*−1/2.
