# Supplementary material for: Crystal structure of 1,8-dibenzoyl-2,7-di­phen­oxy­naphthalene
Source: Acta Crystallogr Sect E Struct Rep Online. 2014 Sep 6;70(Pt 10):170–3. doi: 10.1107/S1600536814019758 (PMC4257173; doi:10.1107/S1600536814019758)
Supplement: Supplementary file 3 [file e-70-00170-Isup3.pdf]

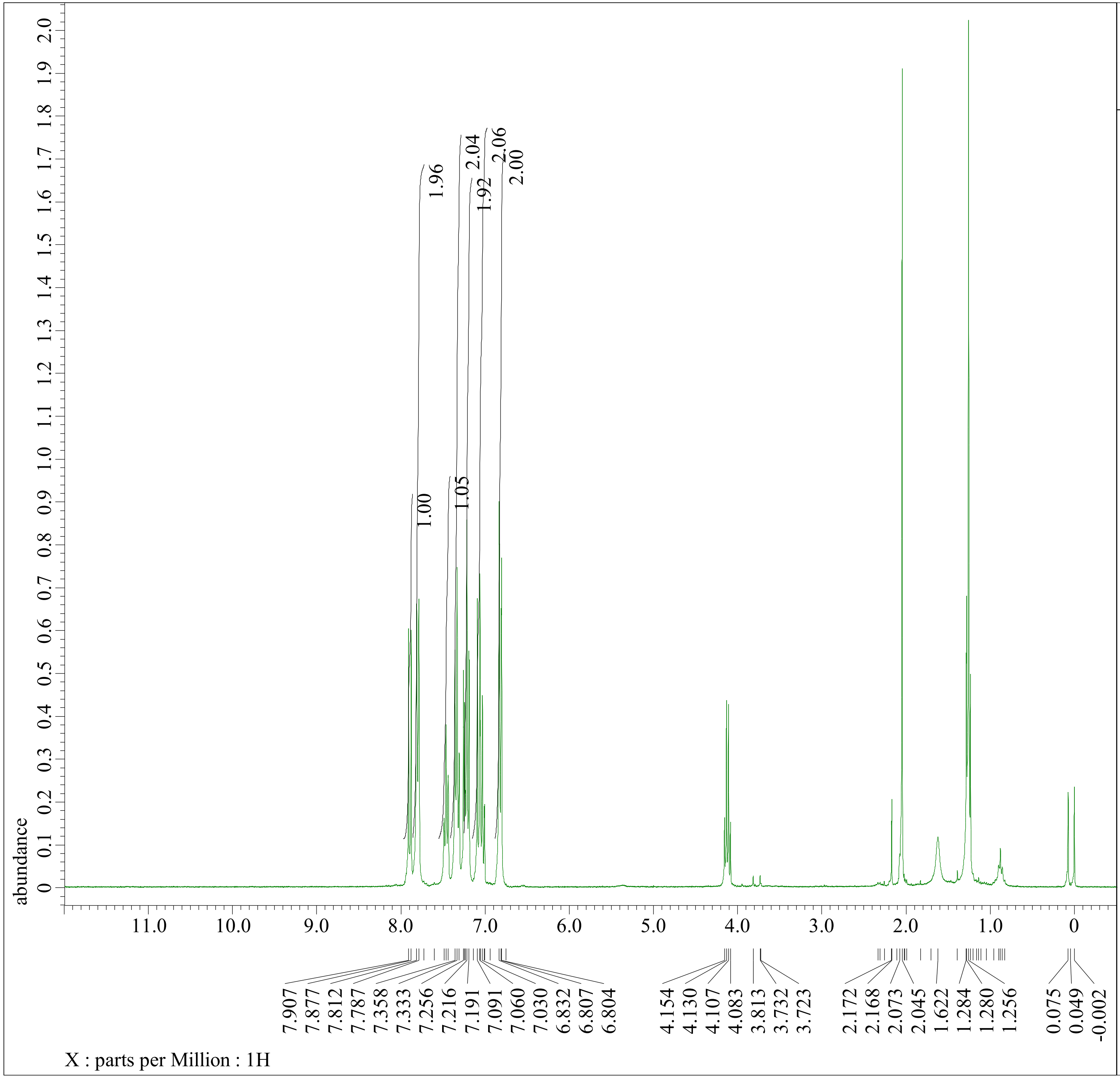

JEOL RESONANCE

Filename	=	140227-narushima-1,8-x-2
Author	=	delta
Experiment	=	single_pulse.ex2
Sample_Id	=	S#656828
Solvent	=	CHLOROFORM-D
Creation_Time	=	27-FEB-2014 17:56:31
Revision_Time	=	30-JUL-2014 19:02:20
Current_Time	=	29-AUG-2014 14:56:00
Comment	=	single_pulse
Data_Format	=	1D COMPLEX
Dim_Size	=	13107
Dim_Title	=	1H
Dim_Units	=	[ppm]
Dimensions	=	X
Site	=	ECX 300
Spectrometer	=	JNM-ECX300
Field_Strength	=	7.0586013[T] (300[MHz])
X_Acq_Duration	=	2.90717696[s]
X_Domain	=	1H
X_Freq	=	300.52965592[MHz]
X_Offset	=	5[ppm]
X_Points	=	16384
X_Prescans	=	1
X_Resolution	=	0.34397631[Hz]
X_Sweep	=	5.63570784[kHz]
Irr_Domain	=	1H
Irr_Freq	=	300.52965592[MHz]
Irr_Offset	=	5[ppm]
Tri_Domain	=	1H
Tri_Freq	=	300.52965592[MHz]
Tri_Offset	=	5[ppm]
Clipped	=	FALSE
Scans	=	4
Total_Scans	=	4
Relaxation_Delay	=	5[s]
Recvr_Gain	=	38
Temp_Get	=	17.2[dC]
X_90_Width	=	17.75[us]
X_Acq_Time	=	2.90717696[s]
X_Angle	=	45[deg]
X_Atn	=	7.06[dB]
X_Pulse	=	8.875[us]
Irr_Mode	=	Off
Tri_Mode	=	Off
Dante_Presat	=	FALSE
Initial_Wait	=	1[s]
Repetition_Time	=	7.90717696[s]
