# Supplementary material for: Crystal structure of 1,8-dibenzoyl-2,7-di­phen­oxy­naphthalene
Source: Acta Crystallogr Sect E Struct Rep Online. 2014 Sep 6;70(Pt 10):170–3. doi: 10.1107/S1600536814019758 (PMC4257173; doi:10.1107/S1600536814019758)
Supplement: Supplementary file 4 [file e-70-00170-Isup4.pdf]

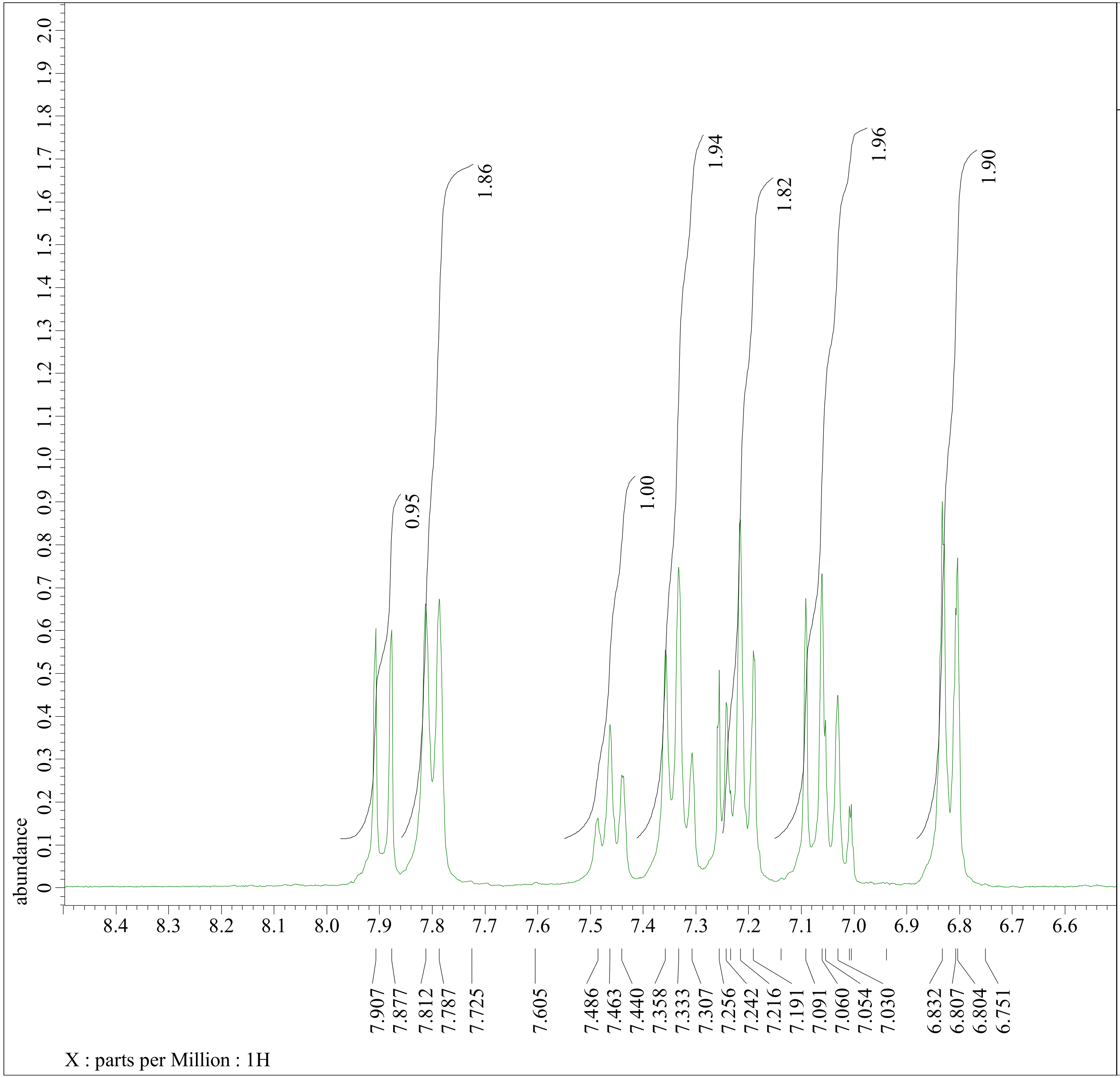

JEOL RESONANCE

Filename	= 140227-narushima-1,8-x-2
Author	= delta
Experiment	= single_pulse.ex2
Sample_Id	= S#656828
Solvent	= CHLOROFORM-D
Creation_Time	= 27-FEB-2014 17:56:31
Revision_Time	= 14-JUL-2014 18:44:37
Current_Time	= 29-AUG-2014 15:16:22
Comment	= single_pulse
Data_Format	= 1D COMPLEX
Dim_Size	= 13107
Dim_Title	= 1H
Dim_Units	= [ppm]
Dimensions	= X
Site	= ECX 300
Spectrometer	= JNM-ECX300
Field_Strength	= 7.0586013[T] (300[MHz])
X_Acq_Duration	= 2.90717696[s]
X_Domain	= 1H
X_Freq	= 300.52965592[MHz]
X_Offset	= 5[ppm]
X_Points	= 16384
X_Prescans	= 1
X_Resolution	= 0.34397631[Hz]
X_Sweep	= 5.63570784[kHz]
Irr_Domain	= 1H
Irr_Freq	= 300.52965592[MHz]
Irr_Offset	= 5[ppm]
Tri_Domain	= 1H
Tri_Freq	= 300.52965592[MHz]
Tri_Offset	= 5[ppm]
Clipped	= FALSE
Scans	= 4
Total_Scans	= 4
Relaxation_Delay	= 5[s]
Recvr_Gain	= 38
Temp_Get	= 17.2[dC]
X_90_Width	= 17.75[us]
X_Acq_Time	= 2.90717696[s]
X_Angle	= 45[deg]
X_Atn	= 7.06[dB]
X_Pulse	= 8.875[us]
Irr_Mode	= Off
Tri_Mode	= Off
Dante_Presat	= FALSE
Initial_Wait	= 1[s]
Repetition_Time	= 7.90717696[s]
