# Supplementary material for: Crystal structure of 1,8-dibenzoyl-2,7-di­phen­oxy­naphthalene
Source: Acta Crystallogr Sect E Struct Rep Online. 2014 Sep 6;70(Pt 10):170–3. doi: 10.1107/S1600536814019758 (PMC4257173; doi:10.1107/S1600536814019758)
Supplement: Supplementary file 5 [file e-70-00170-Isup5.pdf]

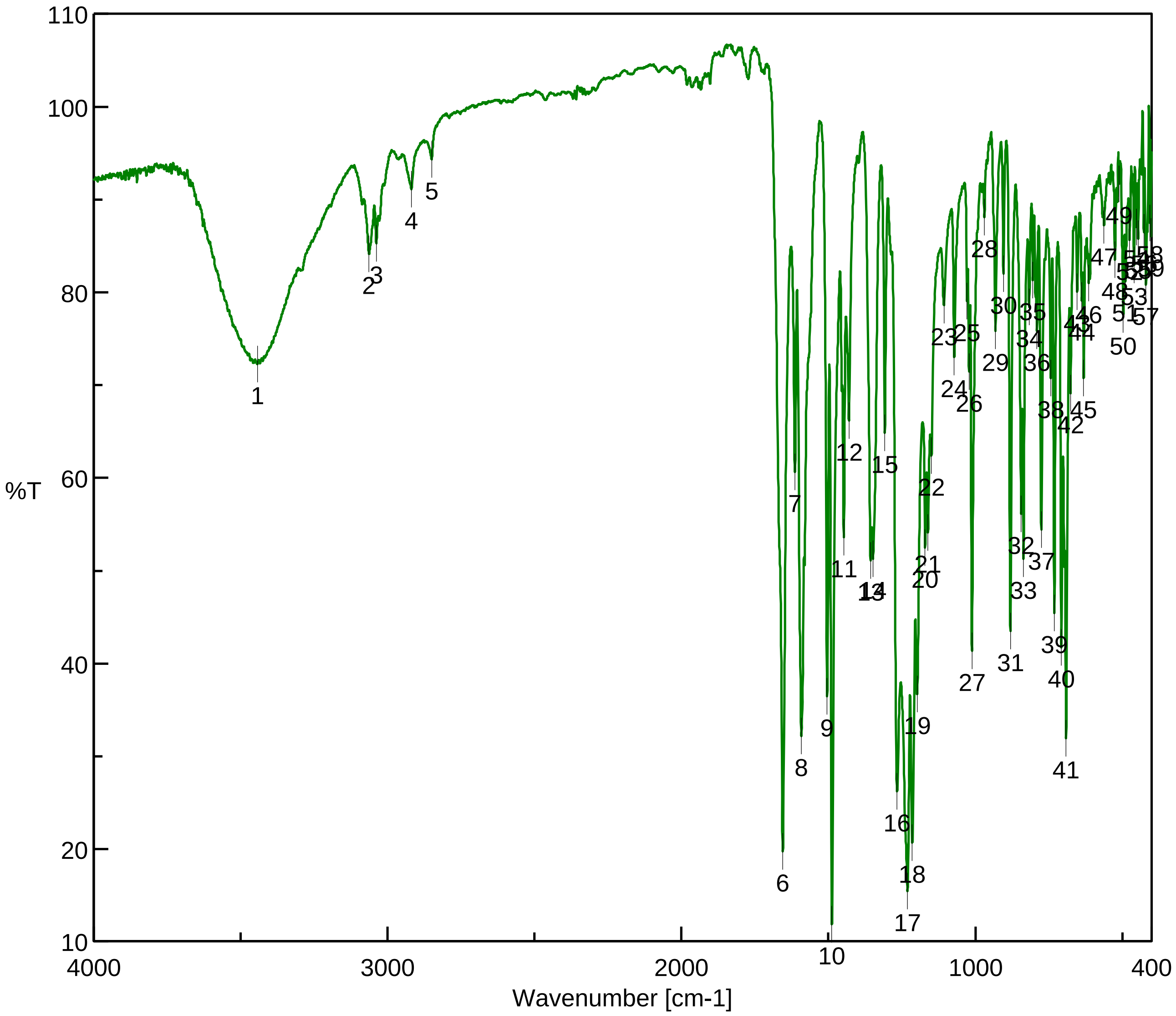

ピーク検出結果

No.	位置	強度	No.	位置	強度
1	3443.28	72.2155	2	3062.89	84.0876
3	3038.3	85.2274	4	2919.22	91.0965
5	2849.79	94.2926	6	1655.11	19.6509
7	1613.64	60.6218	8	1591.95	32.129
9	1504.69	36.3801	10	1488.29	11.8249
11	1447.31	53.5278	12	1429.96	66.1564
13	1356.19	51.0485	14	1348.48	51.2077
15	1307.5	64.7839	16	1266.04	26.123
17	1230.84	15.418	18	1214.45	20.6485
19	1198.06	36.6185	20	1170.58	52.4219
21	1160.94	54.0842	22	1149.37	62.3347
23	1106.94	78.5883	24	1071.75	73.0027
25	1027.87	79.0152	26	1021.61	71.4125
27	1011.48	41.311	28	969.055	88.0652
29	931.45	75.7845	30	904.451	81.9942
31	880.827	43.4548	32	843.704	56.0461
33	835.99	51.2591	34	816.706	78.3753
35	804.171	81.2665	36	790.671	75.8075
37	775.726	54.3528	38	743.424	70.7003
39	730.889	45.3726	40	707.265	41.6522
41	690.873	31.8641	42	675.928	69.0986
43	653.268	80.0024	44	637.84	79.0943
45	631.091	70.7162	46	613.735	80.934
47	561.666	87.2005	48	524.543	83.5019
49	510.562	91.6415	50	496.58	77.5865
51	488.866	81.1297	52	475.367	85.6214
53	459.939	82.9381	54	451.743	86.9788
55	445.958	85.7542	56	426.673	86.4337
57	418.959	80.7813	58	406.907	87.4117
59	402.085	85.9718			

[コメント情報]

試料名	
コメント	
測定者	
所属	
会社	東京農工大学

[測定情報]

機種名	FT/IR-4100typeA
シリアル番号	B041461016
光源	標準光源
検出器	TGS
積算回数	32
分解	2 cm-1
ゼロフィリング	On
アポダイゼーション	Cosine
ゲイン	Auto (16)
アパーチャー	Auto (5 mm)
スキャンスピード	Auto (2 mm/sec)
フィルタ	Auto (30000 Hz)
